# Clinical and gross pathologic findings of complicated vertical fissures with digital dermatitis in a dairy herd

**Published:** 2012

**Authors:** Mohsen Nouri, Javad Ashrafi Helan

**Affiliations:** 1 *Private Veterinary Practitioner, Tehran, Iran;*; 2 *Department of Pathology, Faculty of Veterinary Medicine, University of Tabriz, Tabriz, Iran.*

**Keywords:** Lameness, Granulation tissue, Digital dermatitis, Sand crack, Vertical fissure

## Abstract

Careful antemortem examination and interpretation of findings, assisted by good clinical records, do much to throw light on the nature of vertical fissure in cattle. During an eight month period of investigation, 13 (3.2%) lame cows with vertical fissure out of 52 Holstein cows with different claw fissures were selected for clinical and gross pathological purposes in a commercial dairy farm with 400 milking cows in Nazarabad, Iran. The cows were 2.5 to10.5 years old. The prevalence rate of vertical fissure was 3.2 per cent. The prevalence rate of claw lesion in the hind limb (69.2%) was higher than that of fore limb (30.7%). The type of vertical fissures were 4 (38.4%), 5 (23.0%), 2 (23.0%) and 3 (15.3%), respectively. Locomotion scoring assessment of 13 culled lame cows showed score ranged from grade 3 (30.7%) to 4 (61.5%). The herd had endemic digital dermatitis infection with prevalence in the adult herd of over 34.2%. The affected claws were more boxy than normal and the abaxial wall was convex in all directions. The lame cows had typical stance such as hobbyhorse or cross legged stance. This study shows that more research is needed both on the economic impact of vertical fissures in dairy cows and on the microbiological study of *spirochaetes* of the genus *Treponema*. This study recommends that owners of dairy farm should try to control digital dermatitis with preventative herd strategies.

## Introduction

Vertical fissures (VF) or sand cracks are vertical cracks that may extend across the coronary band and continue distally to the solar surface of the dorsal wall of the claw. The crack may originate at any level of the dorsal wall and extend distally for a variable distance.^1^ The lesion is relatively common in western Canada, in a survey of 15 Alberta cow-calf herds, 37.2% of the cows were affected with one or more cracks.^2^ Researchers examined the forelimbs on 1183 cows from 11 representative herds in Alberta and reported a prevalence of vertical fissures of 22.7%.^3^ A survey of 3615 cows in Saskatchewan demonstrated an overall prevalence of vertical fissures of 16.8%.^4^ Although the incidence of this condition is high in mature Canadian beef cows, the prevalence of lameness in affected cattle is approximately low (0.5%).^5^ In the superficial damage, the coronary band heals, horn formation is restored and the vertical fissures progressively grow out from the toe. Deeper vertical fissures reaching the underlying laminae, lead to infection accompanied by pus formation and, because there is little room for expansion, ends up intense lameness.^6^^,^^7^ Vertical fissures can result in severe lameness and is one of predisposing lesion for septic arthritis^1,5^ which can be one of the most common reasons for deep sepsis of digital bones and premature culling.^8^^,^^9^

Digital dermatitis (DD) mostly occurs on the plantar aspect of the rear foot, affecting the skin adjacent to the interdigital cleft or the skin–horn junction of the heel bulbs. Occasionally, lesions are found adjacent to the dew claws or bordering the dorsal interdigital cleft.^1^^,^^5^^,^^10^^,^^11^ Most lesions are circular or oval, have a strawberry-like appearance, are clearly demarcated by borders with longer hair growing around the lesion and have a distinctive odour.^12^ There is no published report on occurrence of digital dermatitis on other anatomic location in dairy cows to the best of the authors' knowledge. 

This study describes the clinical and gross pathologic presentation of vertical fissures with exposed corium in cases of concurrent digital dermatitis in an endemically infected herd without preventative herd strategies for DD.

## Materials and Methods

Over a period of eight months investigation (January to July 2010), in a commercial dairy farm with 400 milking cows in Nazarabad, Iran, 13 lame cows with vertical fissure out of 52 Holstein cows with claw fissure were selected for clinical and gross pathological purposes. The cows were from a dry lot dairy and fed on a total-mixed ration. The diet consisted of alfalfa hay, corn silage and a commercial concentrate. The animals did not have the clinical evidence of any other systemic disease. The cows were 2.5 to 10.5 years old. Overcrowding, poor drainage and accumulation of feces and urine on the floors and the basis of the concrete bunk was obvious in this dairy farm. The locomotion score was determined by an experienced technician. Each animal was observed standing and walking (on a concrete surface whenever possible) using the Sprecher *et al*. locomotion scoring system.^13^ Then, the lame animals were examined in a hoof trimming box and the affected limbs were raised and the claws were trimmed in accordance with the principle of therapeutic foot care^7^ and tabulated the distribution of claw lesions and its type of vertical fissures using the Greenough vertical fissure typing system.^14^ Details were recorded of all cases of lameness in dairy cows. Foot preparation was the same in all cases. The injury was then photographed using a digital camera (model EOS-350D, Cannon, Tokyo, Japan) to evaluate the extent and severity of claw fissures lesions on the basis of granulated area involved. 

## Results

**Fig. 1:**
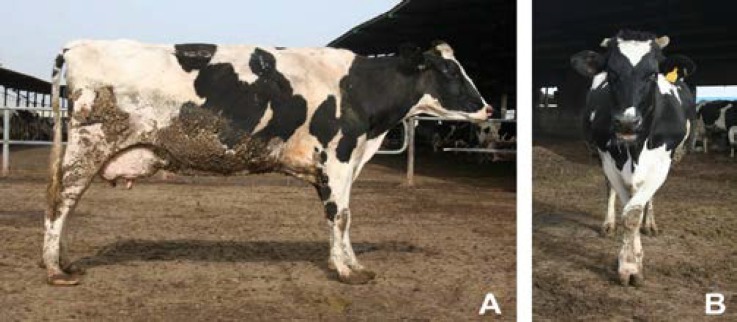
**A.** The lame cows with complicated vertical fissure lesions on the right hind claw shows typical stance such as hobbyhorse. **B.** cross legged stance typical of cow with a vertical fissure on the medial claw of the left forelimb is an effort to take the weight off the medial claws.

All the cases showed signs of abnormal weight bearing. The cows with affected hindlimb had typical stance such as hobbyhorse (Fig. 1A). Some cases with VF on the medial claw of the forelimb had typical cross legged stance (Fig. 1B). Locomotion scoring assessment of 13 culled lame cows showed score ranged from grade 3 (30.7%) to 4 (61.5%) on a scale of 1 to 5.

**Fig. 2:**
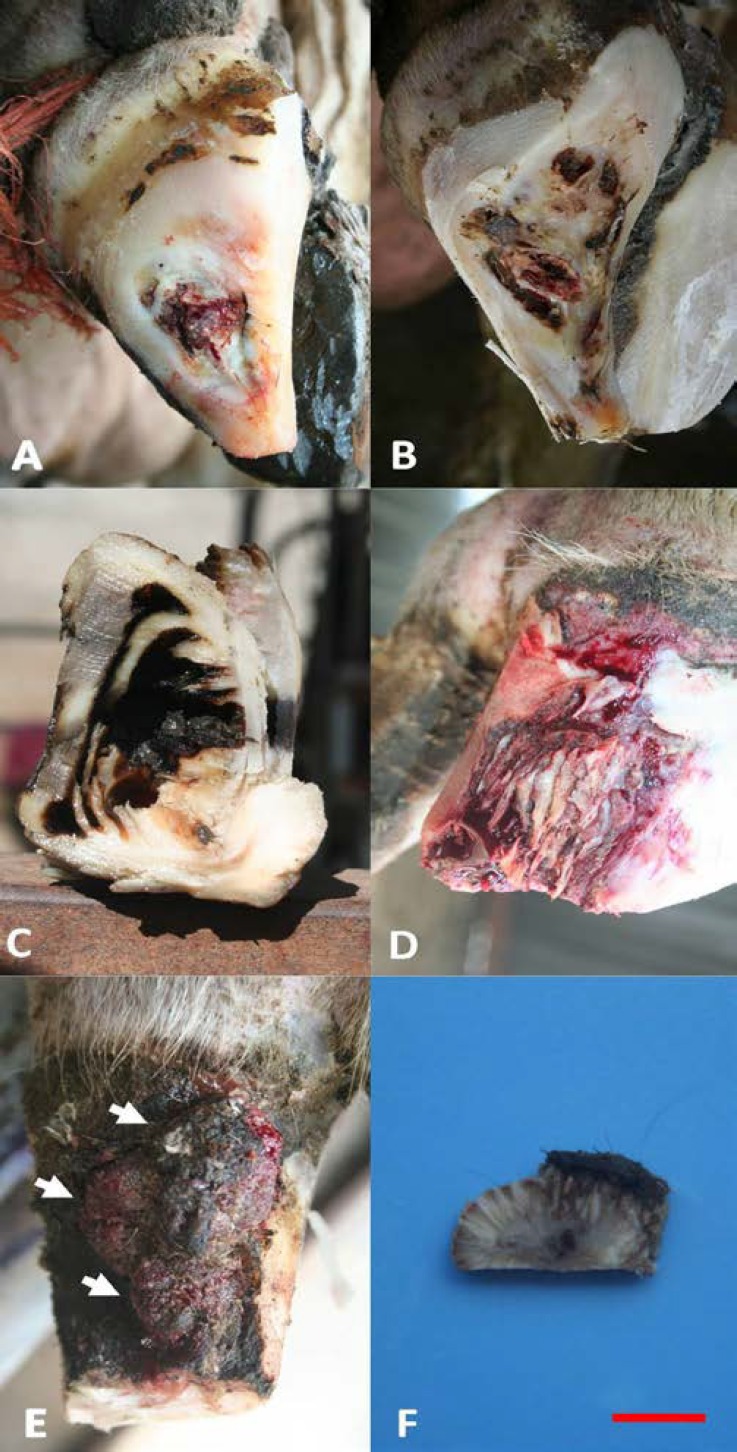
**A. **Type 5 VF involving only the central region of the claw.** B.** Dorsal extent of the lesion; extensive tissue loss in the dorsal region and under-running of the sole.** C.** caudal cross section of the inner surface of the tip of the claw; extensive keratinization tissue loss in the dorsal and toe regions in laminated form.** D.** The same case in photo C showing dorsal extent of the lesion. Lamellar corium of the wall is dry, yellowish, hypertrophic and irregular. **E.** a refractory case; granulation tissue is exuberant and affected by digital dermatitis lesion.** F. **Perpendicular section surfaces reveal that the lesion is a broadly pedunculated hemispherical mass consisting of dermal fibrous connective tissue supporting a convex plaque of uniformly thickened epidermis (the long hairs on the granulation tissue are artificial). Scale bar = 5 mm.

Fifty two cases showed claw fissures (13%); the prevalence rate of affected case with vertical fissures was 3.2%. Table 1 illustrates distribution of vertical fissure in the claws and limbs of dairy cows. The prevalence rate of claw lesion in the hind limb (69.2%) was higher than that of the fore limb (30.7%). The prevalence rate of the types of VF were 4 (38.4%), 5 (23.0%), 2 (23.0%) and 3 (15.3%), respectively. The most common site of vertical fissures was at the lateral hind claw at the center of the claw to the bearing surface (Fig. 2A). The herd had endemic digital dermatitis infection with prevalence in the adult herd of over 34.2%. There was not any sign of other non-infectious lesions associated with vertical fissure on the claw capsule.

**Table 1:** Distribution of vertical fissure (sand crack) in the claws and limbs of the dairy cows.

**Limbs**	**Claws**	**Sand crack**		**Total**	
		**No.**	**%**	**No.**	**%**
**Forelimb**	**Lateral Claw**	1	7.6	4	30.7
	**Medial Claw**	3	23		
**Hind limb**	**Lateral Claw**	7	53.8	9	69.2
	**Medial Claw**	2	15.3		
**Total**	**Lateral Claw**	8	61.5	13	
	**Medial Claw**	5	38.4		

The lesions ranged from impaired horn production, with discoloration, hemorrhages, inferior horn and superficial cracks in the horn of the wall to (eventually) the formation of deeper cracks with variable distance and granulation tissue associated with DD lesions in the wall. The affected claws with VF were more boxy than normal and the abaxial wall was convex in all directions. However, common to all lesions was proliferation of the affected corium and extension along the lamellae along the dorsal wall toward the coronary band. There were extensive tissue loss in the dorsal region and under-running of the sole (Fig. 2B). The solar surface of the affected claw was slightly angled. Cross section of the tip of the claw capsule showed extensive keratinization tissue loss in the dorsal and toe regions in laminated form (Fig. 2C). Lamellar corium of the wall was dry, yellowish, hypertrophic and irregular (Fig. 2D).

In some cases, exuberant granulation tissue was observed. Granulation tissues had a strawberry- like appearance and most of them were hemorrhagic (Fig. 2E). A cross section of the granulation tissue revealed that the lesion is a broadly pedunculated hemispherical mass consisting of dermal fibrous connective tissue supporting a convex plaque of uniformly thickened epidermis exactly such as digital dermatitis lesions (Fig. 2F). 

## Discussion

Vertical fissures occur on dorsal surface of a claw where the granulation tissue was exuberant. Each time the cow bears weight on the affected claw, the two edges of cracks will be pulling or pushing sensitive lamellae in directions. This will be painful and eventually stimulate the formation of granulation tissue. In some refractory cases, particularly those where granulation tissue is exuberant, claw amputation is the only practical option to permanently solve this condition.^7^ In refractory cases of this study, infected granulation tissue was found with digital dermatitis. These lesions are ultimately life-threatening as they may cause extensive damage with severe pain and lameness. A prompt identification and appropriate therapeutic medications can prevent progression of the condition and premature culling.^9^ In this study, all cases did not respond to common therapeutic intervention due to complications of granulation tissue with DD lesions. 

The causes of vertical fissures have not been determined. There has been speculation about the significance or relative importance of several possible contributory factors including trauma, laminitis, hoof size, trace element deficiencies and the mechanical stresses associated with horizontal growth arrest lines, dehydration and exacerbated by shear forces.^1^^,^^3^^,^^14^^-^16 On the other hand, longer term exposure to slurry (up to 14 days) can increase the size and depth of pre-existing cracks and fissures in poor quality hooves,^17^ and this could lead to deeper invasion of harmful environmental and infectious agents such as spirochaetes of the genus *Treponema*.^18^^-^^20^

Although the incidence of this condition is high in mature Canadian beef cows (20%), the prevalence of lameness in affected dairy cattle is approximately low (0.5%).^5^ In this study, the prevalence rate of vertical fissure was 3.2% in dairy cows. Vertical fissures were predominantly found on the lateral claw of the forelimbs in several other surveys.^2^^-^^4^^,^^16^^,^^21^ In our study, this finding does not agree with those of other workers. The reasons for this disagreement are not obvious, but it is probably due to a biomechanical effect caused by weight distribution and ground contact area^3^ and differences in the size of medial and lateral claws, the degree of pronation or supination of the limb, or some other quirk of conformation^14^ and poor management. 

A substantial part of the animal's weight is naturally suspended from the claw wall. The corium is confined within the narrow space of a few millimeters that separates the rigid third phalanx and the horn capsule.^22^ Circulatory disturbances in the corium lead to structural changes (degeneration at the dermal-epidermal junction) in the carrying layers. Eventually the lamellar junction is disrupted and the wall separated from the corium.^22^ In affected claws, the corium of the wall regions was hypertrophic. Horn was yellow and in some cases friable, occurred very commonly in abaxial wall. Normal lamellar corium has pink parietal. ^22^ These changes are at least partially clue to serous fluid being incorporated into the horn and are the principal signs of chronic subclinical laminitis.^22^

Bending of the claw (concavity of the dorsal surface) usually occurs when a vertical fissures is present. It may be associated with a fault (horizontal fissures) or may occur because the tensile strength of the claw has diminished.^14^^,^^22^ Widening of the laminar zone may be caused by accumulation of fluid, blood or cell debris and subsequent separation at the dermal-epidermal junction of the wall; or separation of the same cell layers due to sinkage and hyperplasia of the laminae in the chronic phase.^22^ Once the involved regions in the wall have grown down to the weight-bearing surface, they appear broadened. ^22^

Daily supplementation of the diet with biotin in dairy cows has been shown to reduce lameness problems.^21^^,^^23^A clinical trial showed that primiparous dairy cows supplemented with oral biotin had a decrease in prevalence of white line separation lesions.^24^ This is not to suggest that biotin is a cure for vertical fissures, but rather that it is an agent to reinforce the effect of correcting errors in management.^5^ In the course of study, the role of trace elements was investigated; the major criterion used by dairy producers to select a product was its low price. Although much is known about the role of micronutrients, there is very little information available to guide producers or veterinarians regarding appropriate formulations.^14^

This study shows that more research is needed both on the economic impact of vertical fissures in dairy cows and on the microbiological study of *spirochaetes* of the genus *Treponema*. This study recommends that owners of dairy farm should try to control digital dermatitis with preventative herd strategies.
